# Analyzing Verrucopapillary Lesions of the Oral Cavity: Retracing the Clinicopathological Conundrum

**DOI:** 10.1155/ijod/6627421

**Published:** 2025-10-31

**Authors:** Neetu Jain, Shashi Keshwar, Ashish Shrestha, Navin Agrawal

**Affiliations:** ^1^Department of Oral Pathology, College of Dental Surgery, B. P. Koirala Institute of Health Sciences, Dharan, Nepal; ^2^Department of Conservative Dentistry and Endodontics, College of Dental Surgery, B. P. Koirala Institute of Health Sciences, Dharan, Nepal

**Keywords:** biopsy, prognosis, squamous cell carcinoma, verrucous carcinoma

## Abstract

**Introduction:**

In oral cavity, verrucous papillary lesions (VPLs) can be challenging to diagnose both clinically and histopathologically as they share common overlapping features. The most reported VPLs are verrucous carcinoma (VC), verrucous hyperplasia (VH), and squamous papilloma (SP). Inaccurate diagnosis may lead to suboptimal patient outcomes.

**Objective:**

To evaluate and compare clinicopathological features of reported VPLs to support accurate diagnosis and patient management.

**Materials and Methods:**

10-year retrospective archival with the histopathologic diagnoses of VPLs was taken. A detailed examination of clinical and histopathological data was compared.

**Results:**

VC, followed by VH and SP, were the most reported cases. The koilocytic appearance in all cases points towards the viral association of SP. On the other hand, VH and VC seemed to be habit related. It was easy to diagnose SP and verruciform xanthoma (VX). However, VH and VC presented some overlapping features. Also, variations from ideal characteristics were evident in cases of VC.

**Conclusion:**

The study emphasizes the importance of an accurate biopsy and the accurate diagnosis of benign to malignant VPLs lesions for better treatment and prognosis for the patient.

## 1. Introduction

In the oral cavity, verrucous papillary lesions (VPLs) can range from benign to potentially malignant to frankly malignant, making diagnosis difficult [1]. These lesions are difficult to diagnose both clinically and histopathologically [2]. VPLs of the oral mucosa typically manifest as elevated, whitish, or pinkish masses in the mucosa with either a papillary or verrucous surface. [3] Verruca vulgaris, condylomas, and the more prevalent squamous papilloma (SP) are examples of oral VPLs with a viral etiology. Oral VPLs that are histologically benign typically have a maximum dimension of less than 10 mm and are not very difficult to diagnose [1, 3]. A higher risk of malignant transformation is present in larger oral VPLs that measure 10 mm or more [3]. Verrucous hyperplasia (VH) and proliferative verrucous leukoplakia (PVL) are examples of potentially malignant oral VPLs, whereas malignant lesions include carcinoma cuniculatum, papillary squamous cell carcinoma (SCC), and verrucous carcinoma (VC) [1]. The term “pertaining to or marked by a wart-like growth pattern” describes verrucous lesions. Simplified, verrucous refers to any exophytic or raised growth on the skin's surface or any organ. Not all verrucous lesions are related to the human papillomavirus (HPV), despite popular belief. Some appear to be warts at first glance, but their behavior and prognosis differ greatly. Histopathology is essential for distinguishing between them. [4] A sufficient biopsy and clinical data are necessary for a precise diagnosis. The rationale behind this study is that although VPLs are regularly encountered cases, still there lies the diagnostic dilemma. This problem is due to the overlapping features. Although proper categorization is provided in literature for different VPLs, we tried to confirm it through our study. In this study we observed that VH and VC have particularly overlapping features. Hence, we tried to bring into light these features for a proper diagnosis. As misdiagnosis can result in inappropriate treatment. The objective of this study is to review and categorize VPLs based on clinicohistopathological correlation and the varied appearance in VH and VC.

## 2. Materials and Methods

This is a combined 10-year retrospective study conducted in the department of oral pathology at the B.P. Koirala Institute of Health Science, Dharan, Nepal. The study has been approved by the institutional research committee (IRC/2649/023). The records of the patients with the pathologic diagnoses of VPLs in the study period extending from 2014 to 2023 were retrieved and reviewed only on incisional biopsies with an exophytic growth pattern. The study included only lesions of oral mucosal origin. A total of 102 such cases were recorded, out of which only 89 cases fulfilled the inclusion criteria. The ultimate histological diagnosis was examined and matched with the clinical diagnosis. Only sections stained with hematoxylin and eosin were examined using a light microscope for histological analysis.

Three subject experts of oral pathology first individually analyzed all the slides and categorized the lesions based on the above-mentioned features and thorough literature review. The common consensus cases were first included in the study. While the cases where there were discrepancies among the observers, a consensus meeting was held to reobserve the slides and discuss the findings in detail. Thus, the discrepancies were solved based on collective discussions and mutual agreement. No such methods like diagnosis based on majority vote were considered. This helped all the diagnosis to have undisputed expert consensus. The cases were diagnosed as follows:

1. Malignant: VC
2. Potentially malignant: VH
3. Benign/viral/reactive:

a. SP

b. Verruciform xanthoma (VX)

*Inclusion criteria:* a. The lesions histopathologically presented a verrucous or papillary growth and histopathologically diagnosed as either of the above with characteristic features of the lesions.

b. Cases with complete clinical history

*Exclusion criteria:* a. Verrucopapillary lesion clinically or histopathologically diagnosed as oral SCC.

b. Cases with an incomplete clinical history

c. Recurrence cases

d. Cases with insufficient biopsy sample

e. The histopathological slides with insufficient histopathological features. Example: absence of normal epithelium in case of VC and VH

The criteria followed for conforming to the diagnosis of the lesions were as follows:

1. VC: Verrucous epithelial projections with abundant parakeratin production. Both exophytic and endophytic growth, with broad and elongated rete ridges showing pushing borders into the connective tissue beneath.
2. VH: Epithelial hyperplasia with parakeratosis or hyperkeratosis, a verrucous surface and a certain degree of epithelial dysplasia. No endophytic growth of the hyperplastic epithelium into the lamina propria compared with the adjacent normal mucosal epithelium.
3. SP: Papillary growth lined by stratified squamous epithelium with a connective tissue core.
4. VX: Exophytic growth pattern with defined borders peripherally and deeply. Presence of xanthoma cells within the limits of rete ridges within the connective tissue.

## 3. Result

On a thorough evaluation of 102 cases from archives, 13 cases were excluded due to incomplete history, an inappropriate (inadequate size/lack of normal epithelium) biopsy size. Thus, 89 cases (Table 1) were included in the study, among which more than 60% of cases were VC and less than 6% cases were reported to be VX.

Clinical features: (Tables 2–6)

As per the observation in Table 2, VC is seen to be mostly affecting the people of age group 51–70 years and 31–50 years. Here the *p*-value is significant as per ANOVA suggesting the maximum occurrence of VC at 51–70 years and 31–50 years. The prevalence seemed bimodal in case of SP. However, no significant association between age and other VPLs were evident.

Here in Table 3, Fisher's exact test was applied for all lesions due to low expected counts. The results of the Fisher's exact test suggest the *p*-value is significant (0.018) in case of gender distribution in SP (around 62% of cases are seen in females). There is no such relation between gender and these lesions as per Fisher's exact test. However, in this study all other VPLs seems to be more common in males, for VC, VH, and VX it is around 70%, 80%, and 66%, respectively.

Here in Table 4, as per the results of Fisher's exact test *p*-values suggest that VC shows strong predilection for buccal mucosa followed by gingiva, gingivobuccal sulcus (GBS), lip, and labial mucosa. Also, SP shows to be mostly affecting the sites like palate. Although statistically nonsignificant, the maximum number of cases of VH is noted in buccal mucosa. However, no direct relation between sites and VX is evident.

Here as per Table 5, based on Fisher's exact test, it suggests that there is no association between VPLs and size except in case of VC where a strong association is seen, suggesting most of the lesion were within 1–3 cm.

Here Fisher's exact test in Table 6 suggests that no association is there between most of the VPLs and habits. However, a strong relation is there between tobacco chewing and VC. Although statistically not significant, 50% of the cases of VH were associated with tobacco chewing.

Here Fisher's exact tests in Table 7 show VC is strongly associated with endophytic growth, pushing borders, and absence of dysplasia/koilocytes. VH is defined by epithelial dysplasia and absence of VC-specific features like endophytic growth and pushing borders. SP is related to koilocytes, fibrovascular core, and hyperplastic epithelium.

Here chi-square test in Table 8 shows that the *p*-value is significant in all cases, suggesting that bud-like extension, dysplastic features, and foci of oral squamous cell carcinoma (OSCC) within the epithelium all occur less frequently than the classical VC with ideal presentation.

## 4. Discussion

One of the most difficult and biologically diverse spectrums seen in oral pathology is the collection of lesions referred to as oral VPLs. The first documented evidence of a VPL was a case of well-differentiated oral SCC called “papillary verrucoid carcinoma,” which was reported by Fridell and Rosenthal in 1941 [5]. Different oral VPLs may have similar clinical profiles, but their histopathologic profiles differ. Discriminatory histopathological diagnosis of these lesions is still the “gold standard” and necessitates the presence of normal margins, even though immunohistochemical panels have been considered as adjuncts for the diagnosis of difficult cases [6]. By analyzing 89 well-documented cases of VPLs (Table 1) from institutional archives, our study aimed to shed light on some of these issues. We focused on the importance of morphological variants of VC as well as clinical, histopathological, and etiological factors.

**Figure 1:**
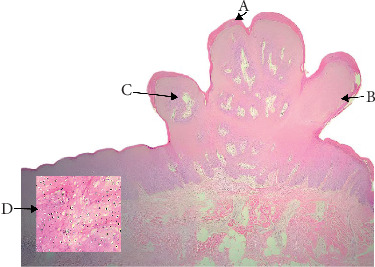
Squamous papilloma (A: keratinization, B: papillary projection, C: connective tissue core, and D: koilocytes).

HPV, particularly subtypes 6 and 11, has been closely linked to SP, one of the most prevalent benign epithelial proliferations of the oral cavity. [7] About 18% of the cases in our study were SP (Table 1). In terms of statistics, SP revealed a significant female predominance (62%) on Fisher's exact test (*p*=0.018) (Table 3). In this study, the palate had the highest concentration of SPs, followed by the tongue and buccal mucosa (Table 4). This result is consistent with findings of Thompson et al. [8] and Chen et al. [7] that describes SP as occurring intraorally, with a preference for keratinized mucosa like the palate. SP lesions in our study were generally less than 1 cm in size, though there were a few instances where they were larger than 1 cm (Table 5). Histopathologically, they showed koilocytic atypia, minimal cytological atypia, little keratinization, and papillary exophytic projections with fibrovascular cores (Figure 1, Table 7). These characteristics matched classic descriptions [1, 8, 9]. In contrast to VC or VH, SP has a well-established viral etiology, and our cohort showed no discernible correlation with tobacco or betel quid (Table 6). This distinction highlights how SP's pathogenesis differs from that of other VPLs. Thus, SP is benign, nonrecurrent, and rarely transforms into malignant. Excision by complete surgery is curative. Differentiating it from other papillary or verrucous lesions is crucial from a clinical standpoint because a superficial resemblance could result in needless aggressive treatment.

**Figure 2:**
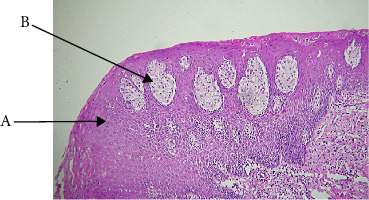
Verruciform xanthoma (A: exophytic growth and B: connective tissue entrapment with xanthoma cells).

VX makes up less than 6% of our cases, that categorize VX as uncommon (Table 1). [1, 9] VX was most prevalent in the fifth and seventh decades and affected both sexes almost equally in our study, and not statistically significant (Tables 2, 3). In clinical settings, VX manifested as pedunculated, sessile, and painless nodules that were typically about 1 cm in size. It's interesting to note that several cases in our study were larger than usual, which could indicate delayed presentation (Table 5). The GBS and buccal mucosa were the most common sites (Table 4), which is consistent with previously published patterns in Philipsen and Reichart. Histologically, VX was easily recognized by its thickened parakeratin, verrucous surface, exophytic growth, hyperplastic epithelium with rete ridges, and—most notably—connective tissue papillae packed with foamy histiocytes. On H nd E, the characteristic eosinophilic color of parakeratin was observed. (Figure 2, Table 7). VX's etiology is still unknown. Potential triggers include inflammation, trauma, or a localized immune response. [1] Our findings, which indicate no correlation with a history of tobacco use (Table 6) or viral infections, lend credence to the lesion's classification as reactive rather than infectious. VX has no potential for malignancy and is benign. Recurrence is uncommon, and total surgical removal is adequate. Its verrucous architecture; however, may clinically resemble VC, underscoring the significance of biopsy.

**Figure 3:**
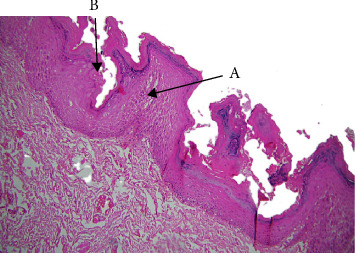
Verrucous hyperplasia (A: exophytic verrucous growth and B: hyperkeratosis).

About 18% of the cases in our study were VH (Table 1), which is like reports from authors like Kallarakkal et al. [2] Similar to VC, VH primarily affected men (80%), peaking in the fifth and sixth decades (Tables 2, 3). Although this was not statistically significant, the buccal mucosa contained the majority of VH lesions (Table 4). VH and VC are believed to be highly interrelated. There is uncertainty regarding the cause of VH and VC. The HPV is thought to be one of the contributing causes. [10, 11] Half of the VH cases in our series were associated with tobacco chewing, although statistical significance was not reached (Table 6). The question of whether VH is causally related to tobacco, betel quid, and HPV infection has long been debated. [10, 11] Patients who chewed one or more betel quid's or smoked one or more cigarettes every day for at least a year were classified as ever chewers or ever smokers by Lee et al. [12]. The etiology of oral VC is complex and dependent on multiple factors [13]. There is evidence of a strong correlation between oral VC and smoking, alcohol consumption, chewing areca nuts, and oral microbiota [14]. These elements may contribute to the development of oral cancer independently or in concert. Oral VC is also associated with chronic inflammation, previous injuries and scars, and undesired prostheses. It may also result from the deterioration of premalignant lesions, such as oral verrucous leukoplakia, oral lichen planus, and oral submucous fibrosis (OSF) [15]. VH may even be an early stage in the pathophysiology of VC, according to authors like kallarakkal et al. [2]. Papillary or verrucous epithelial hyperplasia with dysplasia, lack of endophytic growth, and absence of pushing borders are histological characteristics that characterize VH. Acanthosis, loss of stratification, hyperchromatism, and basal cell hyperplasia were common observations in our study (Figure 3, Table 7). Two subtypes were identified by Shear and Pindborg [16]: sharp type with spiked and highly keratinized processes and blunt type with broad and short projections. In our material, both subtypes were represented. Since they are on the same disease spectrum, VH is frequently thought of as a precursor to VC. It shows epithelial hyperplasia with parakeratosis or hyperkeratosis, a verrucous surface and a certain degree of epithelial dysplasia. No endophytic growth of the hyperplastic epithelium into the lamina propria compared with the adjacent normal mucosal epithelium. In total, 17 leukoplakia have been shown to progress to VH, VC, and ultimately SCC [17]. VH and VC are intermediate stages in the development of PVL to SCC, according to Hansen et al. [18] and Batsakis et al. [19]. The transitional nature of VH lesions was further supported by the atypical features that occasionally bordered on VC in our study.

It is difficult to diagnose oral VC and oral VH lesions both clinically and histopathologically. Shear and Pindborg [16] conducted the first comprehensive histological analysis to attempt to differentiate between VH and VC. In the current study, VC accounted for over 60% of all included cases (Table 1), making it the most common VPL. This result is in line with findings from other studies like Zain et al. [20] where VC is said to be the most prevalent type of VPL because of the region's high prevalence of betel quid and smokeless tobacco use [10]. According to the demographic analysis, VC primarily impacted people between the ages of 50 to 70 (Table 2), and there was a statistically significant correlation between occurrence and age group [21]. Given the lengthy latency period of carcinogenesis linked to long-term exposure to carcinogens like alcohol, tobacco, and areca nut, this age predilection is consistent with the findings of Shear and Pindborg, that characterizes VC as a disease affecting middle-aged and older people [12, 16]. On the other hand, younger patient cases of VC are still uncommon and can frequently be linked to the early onset of addictive behaviors. Nearly 70% of patients in our cohort were male (Table 3), indicating a clear male predominance in VC cases which is like the results of Wang et al. [22]. This result is consistent with previous epidemiological research in South Asian nations, where sociocultural patterns of tobacco and betel quid use account for the high male prevalence. [10, 14, 15] Therefore, although gender is still a statistically significant variable in our study, it most likely reflects sociocultural influences related to habits rather than innate biological predisposition.

Our series' anatomic site distribution revealed that VC was most attracted to the buccal mucosa, gingiva, and GBS (Table 4). This pattern is consistent with research conducted by Franklyn et al. [23] and Alonso et al. [24], which found that the buccal mucosa was the most frequently reported site. This finding has been linked to the habit of placing tobacco or betel quid in the buccal vestibule for extended period of time [12]. On the other hand, recent reports frequently highlight etiological differences by reporting that the larynx and genital mucosa are more common sites of VC. Gingival VC has been linked to diagnostic difficulties, often imitating benign hyperplastic or inflammatory gingival enlargements, which increases the risk of underdiagnosis at initial presentation. Therefore, the preference for gingiva and alveolar mucosa observed in a subset of cases in our study is also noteworthy (Table 4) [16]. Although larger lesions (>3 cm) were not unusual, lesion size analysis showed that most VC lesions measured between 1 and 3 cm at diagnosis (Table 5). In addition to VC's slow growth and locally aggressive nature, the size at presentation also indicates the delay in seeking treatment, which is frequently caused by the condition's seemingly benign verruciform appearance. Because VC frequently manifests as a painless, exophytic, and warty mass, patients and clinicians might underestimate its malignant potential, in contrast to conventional SCC, which may ulcerate or cause significant pain [16, 25]. This emphasizes how crucial it is to be vigilant and get a biopsy as soon as possible for any persistent verruciform lesions larger than 1 cm, especially in high-risk patients.

Perhaps the most significant association observed in this study was between tobacco-related habits and VC. Nearly all VC cases demonstrated a positive history of tobacco use, either in the form of smoking or chewing, with a statistically significant correlation (Table 6). This strongly supports the role of tobacco carcinogens in VC pathogenesis [16, 25]. While tobacco chewing with areca nut has been firmly established as a causative factor, additional etiological contributors such as poor oral hygiene, chronic mechanical irritation, and alcohol consumption have also been implicated in the literature. Moreover, some reports suggest a possible role for HPV, particularly types 16 and 18, in a subset of oral VCs, though the evidence remains inconsistent. [26] Nonetheless, the overwhelming association with tobacco in our study reiterates its primacy as the major risk factor in the South Asian context.

**Figure 4:**
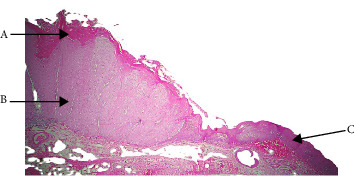
Verrucous carcinoma (A: parakeratin plugging within the cleft like space, B: exophytic and endophytic growth, and C: normal epithelium).

**Figure 5:**
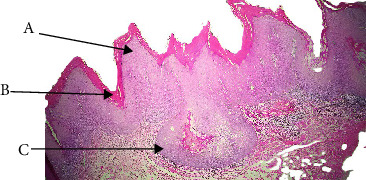
Verrucous carcinoma with classical features (A: exophytic growth, B: keratin plugging with in the cleft, and C: endophytic growth in the form of drop shaped rete pegs).

Histopathologically, VC showed the traditional characteristics that set it apart from other VPLs. These included broad bulbous rete ridges that protruded into the underlying stroma without showing signs of frank invasion, surface clefting, and a noticeably thickened epithelium with prominent parakeratosis. It was common to observe a dense chronic inflammatory infiltrate and keratin plugging within invaginations (Figure 4, Table 7). Given that clinical features alone may overlap with benign hyperplastic lesions or VH, these results validate that histopathology is still the gold standard for diagnosing VC [21]. Our research; however, also reveal the existence of multiple VC variations (Table 8), which have important therapeutic and diagnostic ramifications. The bulk of cases were of the classic form of VC, which was distinguished by its slow growth pattern, broad pushing borders, and lack of cellular atypia (Figure 5). As it rarely spreads and responds well to surgical excision with distinct margins, is typically linked to an excellent prognosis [21]. However, the lesion is locally aggressive and may recur if not properly excised, so the seemingly benign histological appearance should not be misunderstood [21].

**Figure 6:**
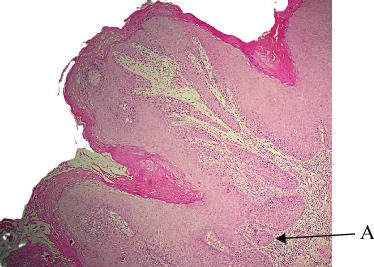
Verrucous carcinoma with bud like extension (A: bud like extension).

Our study found that VC with bud-like extensions (Figure 6) was an important subset. In these instances, focal projections or budding into the underlying connective tissue were seen in otherwise broad, pushing rete ridges. These extensions raise questions about the biological behavior of the lesion because they did not exhibit the cytological atypia typical of invasive SCC. According to published research of Terada et al. [21], these bud-like proliferations could indicate a higher risk of transformation and serve as a transitional stage between VC and traditional SCC. Clinically, this variant must be identified because it may call for broader excision margins and closer monitoring [21].

**Figure 7:**
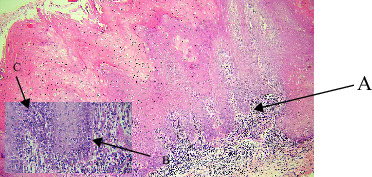
Verrucous carcinoma with dysplastic features (A: basal cell clustering, B: hyperchromatism, and C: mitotic activity).

**Figure 8:**
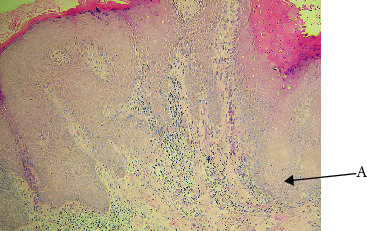
Verrucous carcinoma (A: foci of SCC).

VC with epithelial dysplasia (Figure 7) was another notable variation that was found. The coexistence of epithelial dysplasia raises the possibility of a transitional lesion within the spectrum of VH, VC, and SCC, whereas minimal cytological atypia characterizes classical VC. These cases call into question whether VC always develops from a dysplastic precursor or if it can arise de novo, challenging the traditional histopathological boundaries between these entities [21]. Clinically speaking, dysplasia in VC may indicate a higher risk of malignant development, requiring more aggressive treatment approaches. VC with SCC foci (Figure 8) was arguably the most clinically significant subset reported in our series. These hybrid lesions showed nuclear pleomorphism, mitotic activity, and dyskeratosis, with regions of classic VC morphology interspersed with unmistakable invasive SCC. Since their clinical behavior more closely resembles that of conventional SCC, with a higher risk of recurrence and potential for metastasis, it is crucial to recognize such cases. VC reoccurs locally but doesn't metastasis in the absence of an invasive SCC component [21, 27]. Although excluded from the definition of pure VC, some tumors harbor only dysplasia or minimal invasion, findings of unknown clinical significance. Patel et al. [27] have highlighted that if these hybrid lesions are not identified, they may be treated as pure VC, which may result in less than ideal results [27]. Our results corroborate this theory of Patel et al. [27] and emphasize how crucial it is to obtain sufficient, deep biopsy specimens in order to fully document the lesion's histological spectrum.

Finding VC variants has significant clinical ramifications. Variants with bud-like extensions, dysplasia, or foci of SCC require a more cautious approach, even though classic VC can be effectively managed with local excision and has a favorable prognosis. Depending on the type and severity of the disease, wider surgical excision, potential neck dissection, and even adjuvant radiotherapy may be taken into consideration. Close postoperative monitoring is also necessary to identify progression or recurrences. In addition to diagnosing VC, pathologists are essential in identifying any histological variations in their reports so that proper treatment planning can be guided [21].

Similar biological characteristics of VC and oral SCC include a propensity for local invasion, sneaky lymph node metastasis, and the development of malignant lesions [17]. Both OSCC and VC are associated with tobacco and HPV. Although HPVs are a diverse group of DNA viruses belonging to the Papillomaviridae family, they are clinically significant because they are linked to several cutaneous and mucosal lesions, ranging from benign warts to various cancers. More than 40 distinct HPV types are associated with the clinically most significant genus α-PV, which is further subdivided into low- and high-risk varieties. Low-risk HPV-6 and HPV-11 are primarily linked to benign lesions like anogenital warts and laryngeal papillomas. High-risk HPV-16 is a major contributor to the development of cervical, anal, and vaginal carcinoma as well as a percentage of other cancers, including a subset of SCC of the head and neck [28]. Depending on the product, tobacco may contain over 60 known or suspected carcinogens that can raise the relative risk of cancer through a variety of mechanisms, such as immune system effects, lipids, carbohydrates, and DNA that disrupt cell cycle-regulated mutations, oxidative stress on tissues, and persistent reactive oxygen species [29].

Undoubtedly, the search for trustworthy oral VC molecular markers is essential to overcoming these obstacles. Eight out of the 39 genes were found to exhibit differential expressions between oral VC and oral SCC. These genes include a disintegrin and metalloproteinase with thrombospondin motifs (ADAMTS)-12, α1 type IV collagen (COL4A1), α2 type IV collagen (COL4A2), inhibin βA (INhBA), matrix metalloproteinase 1 (MMP1), serpin peptidase inhibitor clade E member 1 (SERPINE1), transforming growth factor β-induced (TGFBI), and human lactoferrin (hLF) [30]. Biomarkers can also be used to help achieve further differentiation. For example, oral VH, oral VC, and oral SP can be diagnosed with CD34, α-smooth muscle actin, and huR protein. Oral VC and oral SP have comparable morphologies. In the clinical setting, oral SP and oral VC are frequently seen in exophytic, cauliflower, and papillary forms. Histopathologically, oral SP and oral VC can be distinguished from one another. Certain proteins can also be used as markers to accomplish differentiation. The cytokeratin's (CKs) family of proteins, which includes CK10, 13, 14, and 16, is among them. The biological behavior of both SP and VC is correlated with the expression of CKs [31].

For oral VC, surgery has been thought to be the best course of action. Surgery's goal is to remove the tumor without impairing function. Because of the exogenic type of oral VC's controlled size, uncommon tumor recurrence, and favorable prognosis, surgical excision is the first-line treatment option [32, 33]. Also, in our institute, all the cases were treated with surgical excision. On the other hand, surgical excision of the hybrid type of oral VC should be progressive. The hybrid type of oral VC typically has much broader excision sizes, so estimating the excision boundary requires careful consideration. Tumor growth is frequently accelerated by partial or excessive resection, which can result in anaplastic transformation, poor function, and challenging reconstruction. To reduce tumor recurrence and the unfavorable prognosis in this instance, surgery (such as primary tumor resection and neck dissection) in conjunction with radiation and chemotherapy may be suitable [32, 34].

## 5. Conclusion

In conclusion, our study's analysis of VC reveals both its prevalence among oral VPLs and the diversity within the VC spectrum. The etiological role of tobacco is highlighted by the strong alignment of age, gender, site, size, and habit with the body of existing literature. However, the discovery of variations like VC with foci of SCC, VC with dysplasia, and VC with bud-like extensions supports the idea that VC is a morphologically and biologically diverse collection of lesions rather than a single and homogenous entity. To prevent diagnostic errors and guarantee that patients receive individualized, evidence-based treatment plans that strike a balance between oncologic control and quality of life, it is imperative that these variations be recognized.

## 6. Limitation

It would have been more valuable if comparisons between large sample sizes were considered and multicentric studies are conducted. There is possible potential bias in retrospective studies like incomplete records, selection bias and the lack of follow-up data to access outcomes like recurrence or malignant transformation. So long term prospective studies can be a better alternative. Also, the application of molecular profiling for VPLs would have made the study more profound.

## Figures and Tables

**Table 1:** Number of reported cases.

Pathology	Number of cases (*n*)	Percentage (%)
VC	54	60.67
VH	16	17.97
SP	16	17.97
VX	3	3.39

**Table 2:** Distribution of verrucopapillary lesions in different age groups.

Age	Cases											
	VC (54)			VH (16)			SP (16)			VX (3)		
	*n*	%	*p*	*n*	%	*p*	*n*	%	*p*	*n*	%	*p*
<10 years	0	0	1.000	0	0	1.000	2	12.5	0.048	0	0	1.000
11–30 years	0	0	1.000	0	0	1.000	3	18.75	**0.018**	0	0	1.000
31–50 years	24	44.44	**<0.001**	6	37.5	0.770	7	43.75	0.780	2	66.66	0.140
51–70 years	28	51.85	**<0.001**	9	56.25	0.420	3	18.75	**0.018**	1	33.33	1.000
>70 years	2	3.70	0.560	1	6.25	0.530	1	6.25	0.490	0	0	1.000

*Note:* Statistically significant values are marked in bold.

**Table 3:** Distribution of verrucopapillary lesions based on gender.

Cases	Gender					
	Male			Female		
	Number (*n*)	Percentage (%)	*p*-Value	Number (*n*)	Percentage (%)	*p*-Value
VC (54)	38	70.37	0.620	16	29.62	0.620
VH (16)	13	81.25	0.420	03	18.75	0.420
SP (16)	06	37.5	**0.018**	10	62.5	**0.018**
VX (3)	02	66.66	1.00	01	33.33	1.00

*Note:* Statistically significant values are marked in bold.

**Table 4:** Distribution of verrucopapillary lesions based on site of occurrence.

Site^a^	Cases											
	VC (54)			VH (16)			SP (16)			VX (3)		
	*n*	%	*p*	*n*	%	*p*	*n*	%	*p*	*n*	%	*p*
Buccal mucosa	23	42.5	**<0.001**	7	43.7	0.42	1	6.5	0.002	2	66.66	0.42
Tongue	—	—	—	2	12.5	0.69	1	6.5	0.690	—	—	—
Floor of mouth (FOM)	5	9.2	0.320	1	6.5	1.00	2	12.5	0.420	—	—	—
Palate	1	1.8	0.120	1	6.5	1.00	6	37.5	**<0.001**	—	—	—
Gingiva	21	38.8	**<0.001**	3	18.7	0.36	5	31.2	0.180	—	—	—
Gingivobuccal sulcus (GBS)	20	37.0	**<0.001**	1	6.5	0.18	—	—	—	1	33.33	0.69
Lip+labial mucosa	17	31.4	0.002	3	18.7	0.58	1	6.5	0.360	—	—	—

*Note:* Statistically significant values that are less than 0.001 are marked in bold.

^a^Multiple sites were seen to be affected in some patients.

**Table 5:** Distribution of verrucopapillary lesions based on size.

Size	Cases											
	VC (54)			VH (16)			SP (16)			VX (3)		
	*n*	%	*p*	*n*	%	*p*	*n*	%	*p*	*n*	%	*p*
<1 cm	4	7.40	0.486	1	6.25	1.000	3	18.75	0.182	0	0	1.000
1–3 cm	33	61.11	**0.002**	9	56.25	1.000	7	43.75	0.182	1	33.33	1.000
>3–5 cm	15	27.77	0.486	5	31.25	1.000	6	37.5	0.567	2	66.66	0.158
>5 cm	2	3.70	1.000	1	6.25	0.529	0	0	1.000	0	0	1.000

*Note:* Statistically significant values are marked in bold.

**Table 6:** Distribution of verrucopapillary lesions based on habit.

Habit	Cases											
	VC (54)			VH (16)			SP (16)			VX (3)		
	*n*	%	*p*	*n*	%	*p*	*n*	%	*p*	*n*	%	*p*
Tobacco chewing	31	57.40	**0.001**	8	50	0.420	2	12.5	0.002	—	—	—
Tobacco smoking	7	12.96	0.320	4	25	0.690	4	25	0.690	1	33.33	1
Tobacco chewing and smoking	16	29.62	0.120	2	12.5	0.360	3	18.75	0.180	—	—	—
No habit history	—	—	—	2	12.5	0.420	7	43.75	**0.001**	2	66.66	1

*Note:* Statistically significant values that are less than 0.001 are marked in bold.

**Table 7:** Histopathologic findings of verrucopapillary cases.

Histopathologic findings	Cases											
	VC (54)			VH (16)			SP (16)			VX (3)		
	*n*	%	*p*	*n*	%	*p*	*n*	%	*p*	*n*	%	*p*
Keratotic exophytic/papillary	54	100	1.000	16	100	1.000	16	100	1.00	3	100	1.000
Hyperplastic epithelium and acanthosis	54	100	1.000	16	100	1.000	12	75	0.003	3	100	1.000
Endophytic growth	54	100	**<0.0001**	0	0	**<0.0001**	0	0	**<0.0001**	0	0	0.569
Basal cell hyperplasia	54	100	1.000	16	100	1.000	11	68.75	0.013	2	66.66	1.000
Epithelial dysplasia	3	5.55	**<0.0001**	16	100	**<0.0001**	0	0	0.126	0	0	1.000
Pushing borders	54	100	**<0.0001**	0	0	**<0.0001**	0	0	**<0.0001**	0	0	1.000
Keratin plugging	54	100	1.000	16	100	1.000	13	81.25	0.317	2	66.66	1.000
Prominent fibrovascular core	49	90.74	0.317	12	75	0.791	15	93.75	0.003	3	100	0.569
Lymphocytic infiltration in the connective tissue within the vicinity of rete ridges	50	92.59	1.000	10	62.5	0.791	1	6.25	**<0.0001**	3	100	0.108
Koilocytes	5	9.25	**<0.0001**	4	25	1.000	15	93.75	**<0.0001**	2	66.66	0.608
Invasion	0	0	0	0	0	0	0	0	0	0	0	0

*Note:* Statistically significant values that are less than 0.001 are marked in bold.

**Table 8:** Number of cases with VC variants.

Variants	Cases (54)		
	*n*	%	*p*
VC with an ideal presentation	47	87.03	<0.0001
VC with bud like extension into the connective tissue	3	5.55	<0.0001
VC with dysplastic features	3	5.55	<0.0001
VC with foci of oral squamous cell carcinoma	1	1.85	<0.0001

## Data Availability

Data will be made available upon demand.
